# Metabolic dysfunction-associated steatotic liver disease increases hepatocellular carcinoma risk in chronic hepatitis B patients: a retrospective cohort study

**DOI:** 10.3389/fphys.2024.1347459

**Published:** 2024-02-09

**Authors:** Ming Lin, Bowen Gao, Mengnan Peng, Xuefang Chen, Huanming Xiao, Meijie Shi, Xiujuan Zhang, Folai Zeng, Xiaoling Chi

**Affiliations:** ^1^ Department of Hepatology, Guangdong Provincial Hospital of Chinese Medicine, The Second Affiliated Hospital of Guangzhou University of Chinese Medicine, Guangzhou, China; ^2^ The Second Clinical College of Guangzhou University of Chinese Medicine, Guangzhou, China; ^3^ Community Health Service Center of Tianhenan Street Tianhe District, Guangzhou, China; ^4^ Department of Hepatobiliary Surgery, Guangdong Provincial Hospital of Chinese Medicine, The Second Affiliated Hospital of Guangzhou University of Chinese Medicine, Guangzhou, China

**Keywords:** hepatitis B, metabolic dysfunction-associated steatotic liver disease, hepatic steatosis, hepatocellular carcinoma, inverse probability treatment weighting

## Abstract

**Background:** The combined effect of hepatitis B virus infection and metabolic dysfunction-associated steatotic liver disease (MASLD) on hepatocellular carcinoma (HCC) risk remains unclear. The current study sought to elucidate the impact of MASLD on HCC progression in chronic hepatitis B (CHB) patients.

**Method:** This retrospective cohort study included CHB patients who had undergone liver biopsy and abdominal imaging at the Guangdong Provincial Hospital of Chinese Medicine between 2013 and 2019. We investigated the correlation between MASLD and HCC risk, and inverse probability treatment weighting (IPTW) was used to adjust for patient characteristics.

**Results:** A total of 1,613 patients were included, and 483 (29.9%) were diagnosed with MASLD. Over a median follow-up period of 5.02 years, 36 (2.2%) developed HCC, comprising 4.8% (23/483) of those with MASLD and 1.2% (13/1,130) of those without. Those with MASLD had a significantly higher cumulative incidence of HCC than those without (*p* < 0.001). The presence of MASLD was associated with a higher risk of HCC (adjusted hazard ratio [HR], 3.996; 95% confidence interval [CI], 2.007–7.959; *p* < 0.001). After adjustment using IPTW, the patients with MASLD retained a higher cumulative incidence of HCC (*p* < 0.001). Moreover, MASLD was found to be an independent risk factor for the development of HCC (adjusted HR, 10.191; 95% CI, 4.327–24.002; *p* < 0.001). However, among patients with MASLD, there were no significant differences in the cumulative risk of HCC between patients with and without overweight, between those with <2 and ≥2 cardiometabolic risk factors (CMRFs), between those with <3 and ≥3 CMRFs, or between those with <4 and ≥4 CMRFs (*p* = 0.110, *p* = 0.087, *p* = 0.066, and *p* = 0.490, respectively).

**Conclusion:** The presence of MASLD is associated with a higher risk of HCC in patients with CHB. Notably, this higher risk is present in patients with MASLD, irrespective of the presence or absence of overweight or the number of CMRFs they have.

## Introduction

Hepatitis B virus (HBV) infection is a significant global public health issue. In 2019, chronic hepatitis B (CHB) affected about 296 million individuals worldwide, 820,000 of whom died from HBV-related diseases such as liver failure, cirrhosis, or hepatocellular carcinoma (HCC) (You et al., 2023). While long-term antiviral therapy with nucleos(t)ide analogues like entecavir, tenofovir disoproxil fumarate, or tenofovir alafenamide can reduce the risk of HCC occurrence in CHB patients, this treatment cannot completely eliminate the risk (Kim et al., 2022).

Non-alcoholic fatty liver disease (NAFLD) is another severe global health problem, with a worldwide prevalence of 29.8% and a prevalence in China of 29.2% (Le et al., 2019; Zhou et al., 2019). Because of the lack of a unified definition of NAFLD that incorporates key metabolic characteristics, Eslam *et al.* in 2020 proposed renaming NAFLD as metabolic dysfunction-associated fatty liver disease (MAFLD), defining the condition as hepatic steatosis (HS) with obesity, diabetes mellitus (DM), or other metabolic abnormalities (Eslam et al., 2020a; Eslam et al., 2020b). In 2023, a joint proposal, following the Delphi consensus, was made to redefine NAFLD as metabolic dysfunction-associated steatotic liver disease (MASLD) and include patients with HS and at least one of five cardiometabolic risk factors (CMRFs) (Rinella et al., 2023). While some studies have identified a higher risk of HCC in CHB patients who also suffered from NAFLD (Chan et al., 2017; Kanwal et al., 2018), others have found that HS correlates with a decreased risk of HCC in CHB patients (Li et al., 2021; Mak et al., 2021). These conflicting findings suggest that NAFLD has a complex relationship with CHB, especially after being redefined as MASLD. Although the presence of HS may result in the suppression of HBV viral activity and a reduction in liver damage (Tourkochristou et al., 2022), CMRFs such as DM may increase the risk of HCC (Wang et al., 2012). These findings suggest that the individual components of MASLD may have opposing effects on the clinical manifestations of CHB. In the present study, we assessed the effect of MASLD on the risk of HCC in patients with CHB and compared the differences in the effects of MASLD and NAFLD on the risk of HCC in these patients. Inverse probability treatment weighting (IPTW) was used to adjust for potential confounding factors.

## Materials and methods

### Population

This retrospective cohort study included consecutive CHB patients who underwent liver biopsy and abdominal imaging (ultrasonography, magnetic resonance imaging [MRI], or computed tomography [CT]) at the Guangdong Provincial Hospital of Chinese Medicine from 2013 to 2019. In accordance with the multi-society Delphi consensus statement (Rinella et al., 2023), MASLD was defined as HS, diagnosed histologically or by imaging, with at least one of five CMRFs and no other discernible cause of HS. CMRFs included 1) body mass index (BMI) ≥23 kg/m^2^, 2) fasting glucose ≥5.6 mmol/L or type 2 DM or treatment for type 2 DM, 3) blood pressure ≥130/85 mmHg, 4) plasma triglycerides ≥1.70 mmol/L or lipid-lowering treatment, and 5) plasma high-density lipoprotein (HDL) cholesterol ≤1.0 mmol/L in males or ≤1.3 mmol/L in females, or lipid-lowering treatment. Those with no metabolic parameters and no known cause of their HS were deemed to have cryptogenic steatotic liver disease (SLD). NAFLD was defined by the presence of HS, identified through imaging or histology, with its “non-alcoholic” aspect being reflected in the subsequent inclusion and exclusion criteria (National Workshop on Fatty Liver and Alcoholic Liver Disease, Chinese Society of Hepatology, Chinese Medical Association; Fatty Liver Expert Committee, Chinese Medical Doctor Association, 2018).

A baseline assessment was conducted at the time of liver biopsy. We included individuals who met the following criteria: 1) serum hepatitis B surface antigen positivity for ≥6 months (Terrault et al., 2018), 2) ≥18 years regardless of sex, and 3) undergoing regular abdominal imaging procedures, such as ultrasonography, MRI, or CT. Patients were excluded if they 1) had coexistent other viral infections, including hepatitis C virus, hepatitis D virus, and human immunodeficiency virus, 2) had concurrent autoimmune liver disease, 3) consumed >210 g/week or >140 g/week of alcohol for males and females, respectively, 4) another specific etiology that could cause HS, such as drug-induced liver injury and monogenic diseases, 5) a diagnostic history of malignancy, including HCC, before the index date, 6) a follow-up time <6 months or the occurrence of HCC ≤6 months of the index date, or 7) incomplete clinical data.

The diagnostic criterion for liver biopsy is the presence of steatosis in >5% of hepatocytes (Eslam et al., 2020a). The imaging-based diagnosis of HS includes heightened echogenicity of liver parenchyma, decreased visibility of liver structures, and far-field echo attenuation on ultrasonography, a hepatic-to-splenic attenuation ratio of ≤1.0 based on CT, and a proton density fat fraction values of ≥5% on MRI (Chartampilas, 2018).

### Biopsy assessment

Two specialized and experienced histopathologists assessed all liver biopsy specimens to determine the degree of steatosis (using the Brunt classification) and fibrosis (according to the Scheuer scoring system) (Scheuer, 1991; Brunt et al., 1999).

### Endpoint and follow-up

This study sought to quantify the development of HCC. The date of the liver biopsy was used as the index date. The follow-up duration included time from the index date to the date of HCC diagnosis or the last recorded visit. Most patients regularly underwent HCC surveillance through abdominal imaging and semi-annual to annual monitoring of alpha-fetoprotein (AFP) levels. HCC diagnosis was determined radiologically or histologically according to practice guidelines (Zhou et al., 2023).

### Ethics

This study was conducted in adherence with the ethical principles outlined in the 1975 Declaration of Helsinki and approved by the Institutional Review Board of the Guangdong Provincial Hospital of Chinese Medicine (ZE2023–194-01). Given the retrospective nature of the research, the requirement for informed consent was waived.

### Statistical analysis

When comparing the baseline demographic and clinical characteristics between the two group patients, continuous variables were expressed as median (interquartile range [IQR]) and evaluated using the Mann-Whitney *U* test. The Chi-square test was employed to analyze categorical parameters across the two groups. Kaplan-Meier curves were used to plot the cumulative incidence rates of HCC, with intergroup differences assessed using the log-rank test. Clinicopathological factors were screened using the least absolute shrinkage and selection operator (LASSO) Cox regression (Tibshirani, 1997). These factors were then evaluated using multivariate Cox proportional hazards regression, with a bidirectional stepwise method, to identify independent risk factors for HCC.

IPTW was implemented using propensity scores to account for differences in patient characteristics across groups (Chesnaye et al., 2022). Propensity scores were determined by fitting a logistic regression model that included demographic and clinicopathological characteristics as independent variables and the occurrence of MASLD or NAFLD as the dependent variable. After IPTW, we evaluated the balance of the baseline characteristics of study patients between the groups. Weighted Kaplan-Meier curves were used to plot the cumulative incidence rates of HCC. In addition, weighted LASSO Cox regression and weighted multivariate Cox proportional hazards regression were used to identify independent risk factors for the development of HCC.

Statistical analyses were performed using R statistical software (version 4.2.3, R Foundation for Statistical Computing, Vienna, Austria). A two-sided *p*-value of <0.05 was used to indicate statistical significance.

## Results

### MASLD on HCC risk in CHB patients

#### Characteristics of the study population

**FIGURE 1:**
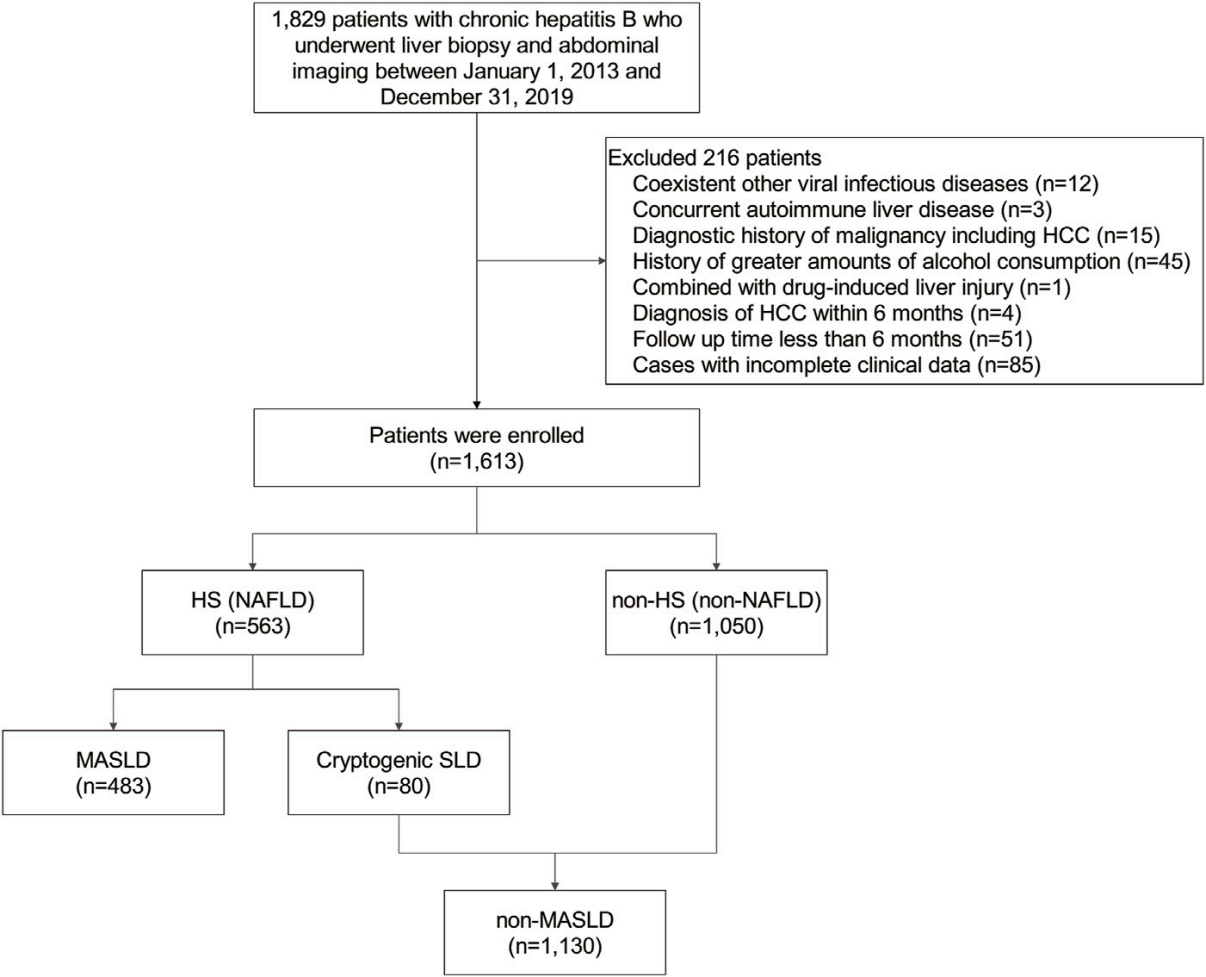
Flowchart of study population selection.

This study included a cohort of 1,613 patients with CHB, 483 (29.9%) of whom were diagnosed with MASLD (Figure 1). Within this MASLD group, HS was confirmed in 448 patients through liver biopsy, in 13 patients via MRI, in 17 patients using CT, and in 5 patients through ultrasonography. The median (IQR) duration of follow-up was 5.02 (3.09–7.12) years. The baseline demographic and clinicopathological features of the subjects under study are outlined in Table 1. Patients in the MASLD group were predominantly older (*p* = 0.002) and males (*p* < 0.001). They exhibited significantly more CMRFs, including overweight, a history of hypertension or DM, increased fasting glucose, triglyceride, total cholesterol, and low-density lipoprotein (LDL) cholesterol levels, and decreased HDL cholesterol levels than patients in the non-MASLD group (all *p* < 0.001). Furthermore, patients in the MASLD group had a longer history of the use of medication, including statins (*p* = 0.018), metformin, and angiotensin-converting enzyme inhibitors (ACEI)/angiotensin receptor blockers (ARBs) (both *p* < 0.001). MASLD patients also had significantly higher serum concentrations of albumin (ALB), alkaline phosphatase (ALP), and gamma-glutamyl transferase (GGT) (all *p* < 0.001), but a lower serum concentration of aspartate aminotransferase (AST) (*p* = 0.014). Interestingly, the MASLD group included fewer patients who had received antiviral treatment for ≥6 months than the non-MASLD group (*p* < 0.001). Notably, the MASLD group had lower baseline levels of HBV DNA and a smaller proportion of hepatitis B e antigen (HBeAg)-positive patients (*p* < 0.001 and *p* = 0.002, respectively).

**TABLE 1:** Baseline clinicopathological characteristics in patients with or without MASLD before and after IPTW.

Characteristics	Before IPTW			After IPTW		
	Non-MASLD (n = 1,130)	MASLD (n = 483)	*p*-value	Non-MASLD (n = 1790.5)	MASLD (n = 1,577.0)	*p*-value
Age (years)			<0.001			0.729
<40	665 (58.8)	217 (44.9)		852.2 (47.6)	716.9 (45.5)	
≥40	465 (41.2)	266 (55.1)		938.3 (52.4)	860.1 (54.5)	
Sex			<0.001			0.542
Female	379 (33.5)	103 (21.3)		494.3 (27.6)	484.4 (30.7)	
Male	751 (66.5)	380 (78.7)		1,296.2 (72.4)	1,092.6 (69.3)	
BMI (kg/m^2^)			<0.001			0.494
<23 (Normal)	834 (73.8)	101 (20.9)		933.8 (52.2)	889.6 (56.4)	
≥23 (Overweight)	296 (26.2)	382 (79.1)		856.7 (47.8)	687.4 (43.6)	
Advanced liver fibrosis			0.475			0.130
No (F0-F2)	857 (75.8)	375 (77.6)		1,344.3 (75.1)	1,050.7 (66.6)	
Yes (F3-F4)	273 (24.2)	108 (22.4)		446.2 (24.9)	526.3 (33.4)	
Diabetes mellitus			<0.001			0.057
No	1,120 (99.1)	433 (89.6)		1757.8 (98.2)	1,517.5 (96.2)	
Yes	10 (0.9)	50 (10.4)		32.7 (1.8)	59.5 (3.8)	
Hypertension			<0.001			0.610
No	1,103 (97.6)	437 (90.5)		1715.8 (95.8)	1,498.2 (95)	
Yes	27 (2.4)	46 (9.5)		74.7 (4.2)	78.8 (5.0)	
Duration of antiviral treatment			<0.001			0.633
Never or <6 months	288 (25.5)	165 (34.2)		435.6 (24.3)	356.2 (22.6)	
≥6 months	842 (74.5)	318 (65.8)		1,354.9 (75.7)	1,220.8 (77.4)	
Duration of statins treatment			0.018			0.689
Never or <1 month	1,108 (98.1)	463 (95.9)		1743.4 (97.4)	1,526.4 (96.8)	
≥1 month	22 (1.9)	20 (4.1)		47.1 (2.6)	50.6 (3.2)	
Duration of metformin treatment			<0.001			0.539
Never or <3 months	1,127 (99.7)	472 (97.7)		1780.3 (99.4)	1,562.9 (99.1)	
≥3 months	3 (0.3)	11 (2.3)		10.2 (0.6)	14.1 (0.9)	
Duration of ACEI/ARBs treatment			<0.001			0.856
Never or <6 months	1,118 (98.9)	458 (94.8)		1752.3 (97.9)	1,540.4 (97.7)	
≥6 months	12 (1.1)	25 (5.2)		38.2 (2.1)	36.6 (2.3)	
Duration of low dose aspirin treatment			1.000			0.696
Never or <3 months	1,127 (99.7)	482 (99.8)		1786.4 (99.8)	1,574.8 (99.9)	
≥3 months	3 (0.3)	1 (0.2)		4.1 (0.2)	2.2 (0.1)	
AFP (ng/mL)			0.651			0.687
≤8.1 (Normal)	902 (79.8)	391 (81.0)		1,402.8 (78.3)	1,205.3 (76.4)	
>8.1 (Elevated)	228 (20.2)	92 (19.0)		387.7 (21.7)	371.7 (23.6)	
Albumin (g/L)	44.60 (41.20, 47.00)	45.30 (42.30, 48.00)	<0.001	43.80 (41.20, 46.70)	43.80 (40.30, 47.50)	0.606
Total bilirubin (μmol/L)	12.90 (9.50, 17.10)	12.50 (9.50, 16.40)	0.196	12.10 (10.10, 16.60)	12.20 (9.40, 17.10)	0.706
ALP (U/L)	72.00 (58.25, 84.00)	76.00 (63.00, 91.00)	<0.001	72.00 (62.00, 87.00)	78.00 (60.00, 92.00)	0.435
ALT (U/L)	37.00 (22.00, 76.00)	40.00 (26.00, 69.50)	0.140	32.00 (19.00, 65.00)	40.00 (26.00, 71.00)	0.054
AST (U/L)	32.00 (23.00, 52.00)	28.00 (22.00, 45.00)	0.014	30.00 (20.00, 45.00)	30.00 (23.00, 54.00)	0.242
GGT (U/L)	27.00 (18.00, 49.00)	35.00 (24.00, 57.00)	<0.001	28.00 (20.00, 51.00)	32.00 (22.00, 52.00)	0.128
Fasting glucose (mmol/L)	4.79 (4.39, 5.09)	5.02 (4.62, 5.56)	<0.001	4.80 (4.54, 5.11)	4.89 (4.49, 5.39)	0.080
Triglyceride (mmol/L)	0.87 (0.69, 1.13)	1.22 (0.94, 1.65)	<0.001	1.00 (0.75, 1.47)	1.01 (0.81, 1.40)	0.934
Total cholesterol (mmol/L)	4.49 (3.98, 5.12)	4.90 (4.21, 5.54)	<0.001	4.73 (4.09, 5.27)	4.65 (3.98, 5.42)	0.554
HDL cholesterol (mmol/L)	1.31 (1.11, 1.53)	1.09 (0.94, 1.27)	<0.001	1.19 (0.96, 1.44)	1.22 (1.00, 1.44)	0.550
LDL cholesterol (mmol/L)	2.78 (2.33, 3.33)	3.22 (2.70, 3.76)	<0.001	2.74 (2.28, 3.41)	2.96 (2.33, 3.52)	0.355
HBV DNA			<0.001			0.493
<6 log_10_ IU/mL	608 (53.8)	316 (65.4)		1,096.9 (61.3)	904.2 (57.3)	
≥6 log_10_ IU/mL	522 (46.2)	167 (34.6)		693.6 (38.7)	672.8 (42.7)	
HBeAg			0.002			0.719
Negative	577 (51.1)	287 (59.4)		1,049.4 (58.6)	891.4 (56.5)	
Positive	553 (48.9)	196 (40.6)		741.1 (41.4)	685.6 (43.5)	

Data were expressed as number (%) or median (IQR).

MASLD, metabolic dysfunction-associated steatotic liver disease; IPTW, inverse probability treatment weighting; BMI, body mass index; ACEI, angiotensin-converting enzyme inhibitors; ARBs, angiotensin receptor blockers; AFP, alpha-fetoprotein; ALP, alkaline phosphatase; ALT, alanine aminotransferase; AST, aspartate aminotransferase; GGT, gamma-glutamyl transferase; HDL, high-density lipoprotein; LDL, low-density lipoprotein; HBV, hepatitis B virus; HBeAg, hepatitis B e antigen; IQR, interquartile range.

#### Development of HCC

**FIGURE 2:**
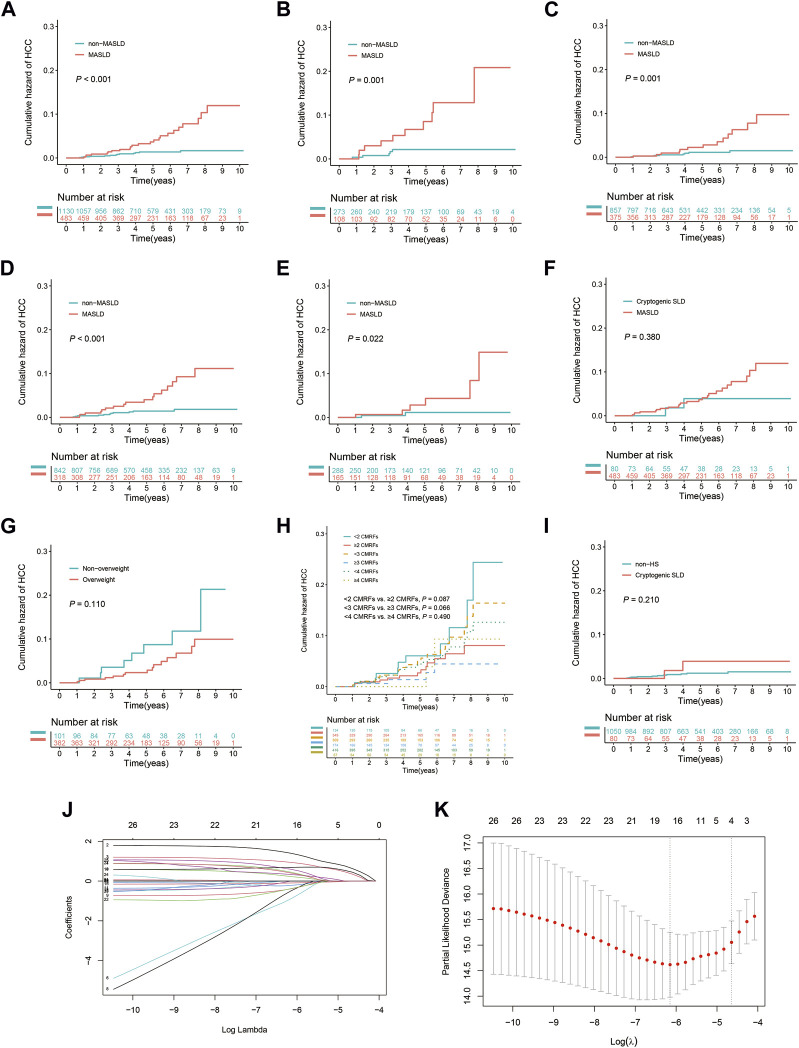
Kaplan-Meier curves and the results of LASSO Cox regression analysis prior to IPTW. **(A)** All patients with or without MASLD. **(B)** Subgroup with advanced liver fibrosis (F3–F4). **(C)** Subgroup without advanced liver fibrosis (F0–F2). **(D)** Subgroup undergoing antiviral treatment for ≥6 months. **(E)** Subgroup never having received antiviral treatment or undergone treatment for <6 months. **(F)** Patients with MASLD or cryptogenic SLD in the HS subgroup. **(G)** Patients with or without overweight (BMI ≥23 kg/m^2^) in the MASLD subgroup. **(H)** Patients exhibit varying levels of CMRFs in the MASLD subgroup. **(I)** Patients with cryptogenic SLD or without HS in the non-MASLD subgroup. **(J)** Selection of risk factors using LASSO Cox regression analysis. **(K)** The four risk factors selected using LASSO Cox regression analysis.

During the follow-up period, HCC was diagnosed in 36 patients (2.2%). Of these, 4.8% (23/483) were in the MASLD group and 1.2% (13/1,130) were in the non-MASLD group. The 3- and 5-year cumulative incidence rates of HCC among patients in the MASLD group were 1.7% and 3.7%, respectively, markedly surpassing the 0.8% and 1.4% rates observed in the non-MASLD group (*p* < 0.001) (Figure 2A). MASLD patients with both advanced liver fibrosis and non-advanced liver fibrosis had a significantly higher cumulative HCC incidence than patients in the non-MASLD group (both *p* = 0.001) (Figures 2B, C). In addition, MASLD patients had a higher cumulative HCC incidence than non-MASLD patients regardless of whether they had received antiviral treatment for ≥6 months (*p* < 0.001 and *p* = 0.022, respectively) (Figures 2D, E). In the HS subgroup, the cumulative risk of developing HCC in patients with MASLD did not differ from that of those with cryptogenic SLD (*p* = 0.380) (Figure 2F). Within the MASLD subgroup, there was no significant difference in the cumulative risk of HCC between patients who had overweight (BMI ≥23 kg/m^2^) or not (BMI <23 kg/m^2^), between those with <2 and ≥2 CMRFs, between those with <3 and ≥3 CMRFs, or between those with <4 and ≥4 CMRFs (*p* = 0.110, *p* = 0.087, *p* = 0.066, and *p* = 0.490, respectively) (Figures 2G, H). In the non-MASLD subgroup, the cumulative risk of HCC in patients with cryptogenic SLD did not differ from that of those without HS (*p* = 0.210) (Figure 2I).

Four risk factors for HCC were identified using LASSO Cox regression when the lambda was set to 1 standard error: age (≥40 years), the presence of MASLD, elevated AFP, and ALB levels (Figures 2J, K). As shown in Table 2, a close association between the concomitant presence of MASLD and a high risk of HCC was identified using multivariate Cox analysis with bidirectional stepwise variable selection (adjusted hazard ratio [HR], 3.996; 95% confidence interval [CI], 2.007–7.959; *p* < 0.001). In addition, the following were identified as independent risk factors for HCC: age (≥40 years) (adjusted HR, 3.185; 95% CI, 1.437–7.057; *p* = 0.004), elevated AFP (adjusted HR, 2.488; 95% CI, 1.211–5.113; *p* = 0.013), and ALB levels (adjusted HR, 0.927; 95% CI, 0.861–0.997; *p* = 0.041).

**TABLE 2:** Multivariate Cox analysis of the factors associated with the development of HCC before and after IPTW.

Characteristics	Before IPTW		After IPTW	
	Adjusted hazard ratio (95% CI)	*p*-value	Adjusted hazard ratio (95% CI)	*p*-value
Age (years)		0.004		
<40	1 [Reference]			
≥40	3.185 (1.437–7.057)			
MASLD		<0.001		<0.001
No	1 [Reference]		1 [Reference]	
Yes	3.996 (2.007–7.959)		10.191 (4.327–24.002)	
AFP (ng/mL)		0.013		
≤8.1 (Normal)	1 [Reference]			
>8.1 (Elevated)	2.488 (1.211–5.113)			
Albumin (g/L)	0.927 (0.861–0.997)	0.041	0.882 (0.827–0.941)	<0.001
HBV DNA				0.004
<6 log_10_ IU/mL			1 [Reference]	
≥6 log_10_ IU/mL			0.168 (0.049–0.574)	
HBeAg				0.023
Negative			1 [Reference]	
Positive			4.309 (1.220–15.225)	

Data in parentheses are 95% CIs.

IPTW, inverse probability treatment weighting; HCC, hepatocellular carcinoma; CI, confidence interval; MASLD, metabolic dysfunction-associated steatotic liver disease; AFP, alpha-fetoprotein; HBV, hepatitis B virus; HBeAg, hepatitis B e antigen.

#### Evaluation of HCC risk after IPTW

**FIGURE 3:**
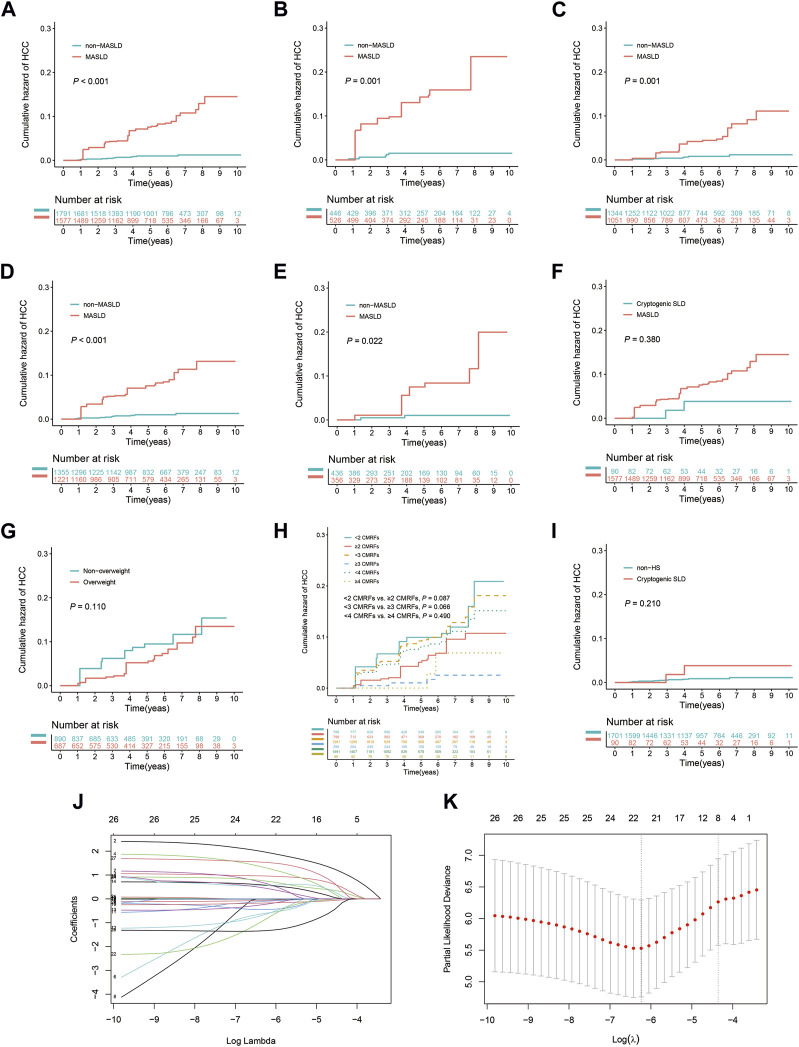
Weighted Kaplan-Meier curves and the results of weighted LASSO Cox regression analysis following IPTW. **(A)** All patients with or without MASLD. **(B)** Subgroup with advanced liver fibrosis (F3–F4). **(C)** Subgroup without advanced liver fibrosis (F0–F2). **(D)** Subgroup undergoing antiviral treatment for ≥6 months. **(E)** Subgroup never having received antiviral treatment or undergone treatment for <6 months. **(F)** Patients with MASLD or cryptogenic SLD in the HS subgroup. **(G)** Patients with or without overweight (BMI ≥23 kg/m^2^) in the MASLD subgroup. **(H)** Patients exhibit varying levels of CMRFs in the MASLD subgroup. **(I)** Patients with cryptogenic SLD or without HS in the non-MASLD subgroup. **(J)** Risk factors identified using weighted LASSO Cox regression analysis. **(K)** The eight risk factors selected using weighted LASSO Cox regression analysis.

After using IPTW to adjust for patient characteristics, baseline demographic and clinicopathological attributes, as well as medication history, including age, sex, BMI, DM, hypertension, lipid profiles, liver function indicators, HBV DNA levels, HBeAg, and the duration of antiviral, statins, metformin, and ACEI/ARBs treatment, were effectively balanced between the groups (Table 1). Consistent with the results obtained prior to IPTW matching, patients in the MASLD group exhibited a 3- and 5-year cumulative incidence rate of 4.3% and 7.5%, respectively, significantly exceeding the 0.6% and 1.0% rates in the non-MASLD group (*p* < 0.001) (Figure 3A). Additionally, patients in the MASLD group had a markedly elevated cumulative HCC incidence rate than those in the non-MASLD group in subgroups with advanced liver fibrosis (*p* = 0.001) and without advanced liver fibrosis (*p* = 0.001) (Figures 3B, C). Likewise, patients in the MASLD group had a significantly higher cumulative incidence of HCC than those in the non-MASLD group regardless of whether they used antiviral treatment for ≥6 months (*p* < 0.001 and *p* = 0.022, respectively) (Figures 3D, E). In the HS subgroup, the cumulative risk of HCC in patients with MASLD did not differ from that of patients with cryptogenic SLD (*p* = 0.380) (Figure 3F). In the MASLD subgroup, there were no significant differences in the cumulative risk of HCC between patients who had overweight and those who did not (*p* = 0.110), between those with <2 and ≥2 CMRFs (*p* = 0.087), between those with <3 and ≥3 CMRFs (*p* = 0.066), or between those with <4 and ≥4 CMRFs (*p* = 0.490) (Figures 3G, H). In the non-MASLD subgroup, the cumulative risk of HCC in patients with cryptogenic SLD did not differ from that of those without HS (*p* = 0.210) (Figure 3I).

Weighted LASSO Cox regression was employed to identify factors significantly associated with the risk of progression to HCC (Figure 3J). The identified factors were age (≥40 years), the presence of MASLD, advanced liver fibrosis, levels of ALB, ALP, and HDL cholesterol, HBV DNA concentration ≥6 log_10_ IU/mL, and HBeAg positivity (Figure 3K). Subsequently, these factors were further evaluated through bidirectional stepwise variable selection in a weighted multivariate Cox analysis, which identified several independent risk factors for HCC: MASLD (adjusted HR, 10.191; 95% CI, 4.327–24.002; *p* < 0.001), ALB levels (adjusted HR, 0.882; 95% CI, 0.827–0.941; *p* < 0.001), HBV DNA concentration ≥6 log_10_ IU/mL (adjusted HR, 0.168; 95% CI, 0.049–0.574; *p* = 0.004), and HBeAg positivity (adjusted HR, 4.309; 95% CI, 1.220–15.225; *p* = 0.023).

#### NAFLD on HCC risk in CHB patients

**FIGURE 4:**
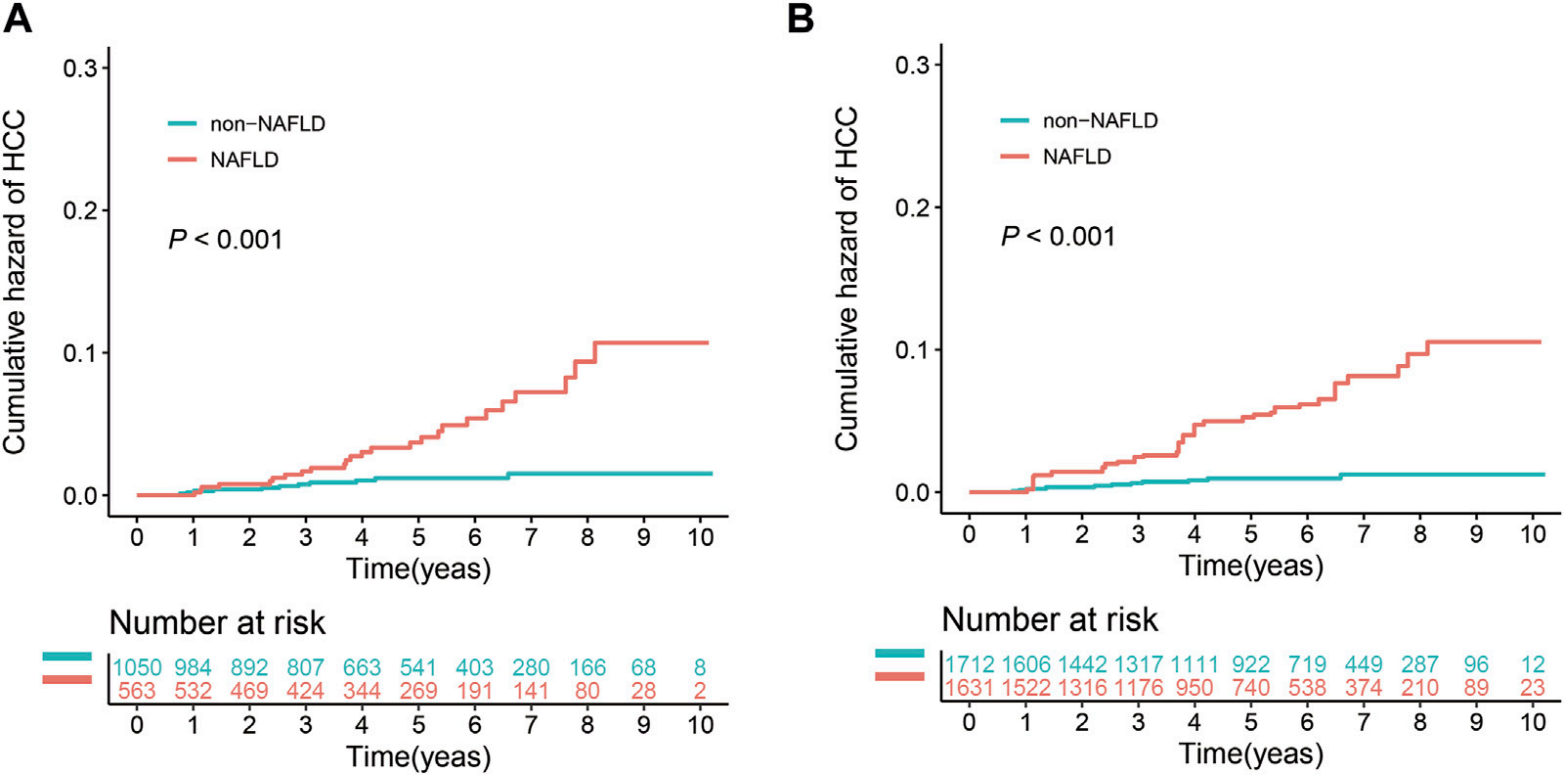
Effect of NAFLD on the cumulative risk of HCC before and after IPTW. **(A)** Cumulative risk of HCC before IPTW. **(B)** Cumulative risk of HCC after IPTW.

During the study, 34.9% (563/1,613) of the patients were diagnosed with NAFLD (Figure 1). The baseline demographic and clinicopathological features of these patients are shown in Supplementary Table S1. The clinical characteristics of the NAFLD and non-NAFLD groups were similar to those of the MASLD and non-MASLD groups. During the follow-up period, 4.4% (25/563) of the patients in the NAFLD group developed HCC, in contrast to 1.0% (11/1,050) in the non-NAFLD group. The 3- and 5-year cumulative incidence rates of HCC in the NAFLD group were 1.7% and 3.7%, respectively, which were significantly higher than the 0.8% and 1.2% in the non-MASLD group (*p* < 0.001) (Figure 4A). After IPTW adjustment, the demographic, clinicopathological features, and history of medication of the two groups were well balanced (Supplementary Table S1). The 3- and 5-year cumulative incidence rates of HCC in the NAFLD group were 2.5% and 5.2%, respectively, which significantly exceeded the 0.6% and 1.0% rates in the non-NAFLD group (*p* < 0.001) (Figure 4B).

## Discussion

This retrospective study evaluated the effect of concurrent MASLD on the incidence of HCC in Chinese patients with CHB. As far as we know, this is the first study to report the correlation between MASLD and the risk of HCC in CHB patients. This study included 1,613 CHB patients, 29.9% of whom had MASLD. This prevalence rate aligns with the MASLD prevalence in the general population as reported in a previous study (Ciardullo et al., 2023). The incidence of CMRFs, including overweight, DM, hypertension, and dyslipidemia, was higher for patients in the MASLD group than the non-MASLD group. Correspondingly, patients in the MASLD group had a longer history of using statins, metformin, and ACEI/ARBs than those in the non-MASLD group. Interestingly, patients in the MASLD group had a lower baseline level of HBV DNA and a reduced likelihood of being HBeAg-positive. Several studies have shown a decrease in viral replication among CHB patients with HS (Wang et al., 2014; Ceylan et al., 2016), yet the underlying mechanisms remain obscure, suggesting a potential interplay between HBV and CMRFs during liver disease pathogenesis (Wang and Xie, 2022). Further study is needed to explore this relationship further.

Multivariable Cox analysis identified MASLD as an independent risk factor for developing HCC. CHB patients with MASLD had a 4-fold increased risk of HCC than those without. Even after adjusting for potential confounders using IPTW score matching, MASLD persistently emerged as an independent risk factor for HCC. Similarly, van Kleef *et al.* reported that CHB patients with MAFLD had a 2-fold higher risk of developing HCC (van Kleef et al., 2021). Another study using the South Korean National Health Insurance System database found that after adjusting for age, gender, cirrhosis, antiviral treatment, physical activity, smoking history, and alcohol consumption, the risk of developing HCC was 1.4-fold higher in CHB patients with MAFLD (Yun et al., 2022). After applying IPTW, there was a persistent association between the risk of developing HCC in CHB patients with MASLD regardless of whether they had advanced liver fibrosis. This suggests that MASLD may contribute to HCC progression independent of liver fibrosis. These findings indicate that patients with non-advanced liver fibrosis and MASLD should undergo increased HCC surveillance. Traditionally, advanced liver fibrosis has been considered a major HCC risk factor (Fujiwara et al., 2018). However, in the current study, it was excluded from the multivariate Cox model. The independent risk factors for HCC identified were age (≥40 years), MASLD, elevated AFP, and ALB levels. Advanced liver fibrosis was excluded even after mitigating potential confounding variables using IPTW. This may be attributed to the limitations of observational studies, including potential overlooked or unknown confounders that could bias the association between advanced liver fibrosis and HCC. However, the findings also suggest that advanced liver fibrosis may not be the only or leading cause of HCC. A broader spectrum of risk factors should be considered during HCC risk assessment and management, including age, MASLD, AFP, and ALB levels.

This study also discovered that the association between MASLD and HCC risk persisted in CHB patients regardless of whether they had received antiviral treatment for more than 6 months, both before and after adjustment with IPTW. The majority of patients in this study had undergone antiviral therapy for ≥6 months, primarily with entecavir or tenofovir disoproxil fumarate. Notably, patients in the MASLD group were more likely to have never received antiviral therapy or received therapy for <6 months than to have received therapy for ≥6 months, probably because this group had lower levels of HBV replication. While antiviral drugs can inhibit viral replication, averting liver disease progression and potentially offering long-term prevention against HCC (Gordon et al., 2014), the current study found that HCC risk remained high in MASLD patients who had received long-term treatment. This is particularly true for patients with HBV DNA levels ≥6 log_10_ IU/mL or a positive HBeAg status, as these were identified as independent risk factors for HCC in CHB patients after adjusting for confounders with IPTW. Thus, in evaluating the risk of HCC in CHB patients who adhere to antiviral therapy, careful consideration should be given to MASLD.

Furthermore, we found that, compared to patients with CHB who had cryptogenic SLD, those with MASLD did not exhibit a significantly higher cumulative risk of developing HCC. Similarly, the cumulative risk of HCC in patients with cryptogenic SLD did not significantly differ from that of the patients without HS. This finding remained after IPTW with respect to the baseline characteristics of the patients. This suggests that the cumulative effect of multiple factors, including CMRFs and HS, mediate the effects of MASLD on the development of HCC in patients with CHB. However, the precise influence of each factor on the risk of HCC requires further investigation. Although the cumulative incidence of HCC was higher in patients with MASLD, those with overweight and those with ≥2, ≥3, or ≥4 CMRFs did not significantly influence the risk of HCC in the present sample of patients. Although overweight and CMRFs have been shown to be associated with a higher risk of HCC (Larsson and Wolk, 2007; Antwi et al., 2022), the present findings indicate that in patients with MASLD, the risk of developing HCC does not differ with varying levels of CMRFs. The findings also imply that in the presence of MASLD, the risk of HCC may also be higher in patients without overweight and in those with few CMRFs, which underscores the need for clinical vigilance.

To compare the risk of HCC in patients with CHB and either the newly named MASLD or the previously named NAFLD, we reclassified the patients according to whether they had NAFLD or not, to evaluate the effect of NAFLD on the development of HCC. The results showed that, both before and after adjustment using IPTW, the cumulative risk of HCC in the NAFLD group was significantly higher than that of the non-NAFLD group. However, after IPTW adjustment, the 3- and 5-year cumulative risks of HCC in the NAFLD group were 2.5% and 5.2%, respectively, which were lower than those in the MASLD group (4.3% and 7.5%). This suggests that the presence of MASLD, which includes a greater emphasis on metabolic parameters, is associated with a higher risk of HCC. This further underscores the importance of metabolic defects in the development of HCC.

The current study has some limitations. Its retrospective design prevented the collection of data on waist circumference, 2-h postprandial blood glucose, and glycated hemoglobin. The absence of this information could have led to an underdiagnosis of MASLD in certain patients and affected the study outcomes. Additionally, the evolving status of MASLD during the follow-up period could have changed the level of HCC risk in certain patients, a consideration not accounted for in the retrospective cohort. This limitation underscores the need for prospective studies to evaluate the correlation between MASLD and HCC risk in CHB patients while concurrently adjusting for the chronicity of MASLD. Finally, while the median follow-up duration was 5.02 years, this may not have been enough time to estimate the full impact of MASLD on HCC risk.

## Conclusion

The present findings highlight the critical role of MASLD as an independent risk factor for HCC in patients with CHB. The findings were consistent when a number of subgroups were analyzed, including patients with and without advanced liver fibrosis and those undergoing antiviral therapy for <6 and ≥6 months. The higher risk of HCC in patients with CHB who have MASLD is driven by the cumulative effect of multiple factors, including CMRFs and HS. Furthermore, when compared to NAFLD, the classification MASLD places greater emphasis on metabolic factors and is associated with a higher risk of HCC. These findings suggest the importance of screening for steatosis, and especially MASLD, to reduce the risk of HCC in patients with CHB. Notably, even patients with MASLD who do not have overweight or have few CMRFs are at a high risk of HCC. Further investigations are needed to elucidate the mechanisms underlying the relationship between MASLD and HCC, which could inform the development of novel strategies for the prevention of HCC in this high-risk population.

## Data Availability

The raw data supporting the conclusion of this article will be made available by the authors, without undue reservation.
