# Supplementary material for: Metabolic dysfunction-associated steatotic liver disease increases hepatocellular carcinoma risk in chronic hepatitis B patients: a retrospective cohort study
Source: Front Physiol. 2024 Feb 9;15:1347459. doi: 10.3389/fphys.2024.1347459 (PMC10886697; doi:10.3389/fphys.2024.1347459)
Supplement: Supplementary file 1 [file Table1.DOCX]

**Supplementary Table S1. Baseline clinicopathological characteristics in patients with or without NAFLD before and after IPTW.**

| **Characteristics** | **Before IPTW** | | |  | **After IPTW** | | |
| --- | --- | --- | --- | --- | --- | --- | --- |
|  | **non-NAFLD**  **(n = 1050)** | **NAFLD**  **(n = 563)** | ***P*-value** |  | **non-NAFLD**  **(n = 1711.7)** | **NAFLD**  **(n = 1631.4)** | ***P*-value** |
| Age (years) |  |  | 0.001 |  |  |  | 0.220 |
| <50 | 881 (83.9) | 435 (77.3) |  |  | 1411.8 (82.5) | 1288.4 (79.0) |  |
| ≥50 | 169 (16.1) | 128 (22.7) |  |  | 299.9 (17.5) | 343.0 (21.0) |  |
| Sex |  |  | <0.001 |  |  |  | 0.387 |
| Female | 355 (33.8) | 127 (22.6) |  |  | 491.4 (28.7) | 521.1 (31.9) |  |
| Male | 695 (66.2) | 436 (77.4) |  |  | 1220.3 (71.3) | 1110.3 (68.1) |  |
| BMI (kg/m^2^) |  |  | <0.001 |  |  |  | 0.355 |
| <23 (Normal) | 754 (71.8) | 181 (32.1) |  |  | 938.2 (54.8) | 962.1 (59.0) |  |
| ≥23 (Overweight) | 296 (28.2) | 382 (67.9) |  |  | 773.5 (45.2) | 669.3 (41.0) |  |
| Advanced liver fibrosis |  |  | 0.297 |  |  |  | 0.458 |
| No (F0-F2) | 793 (75.5) | 439 (78) |  |  | 1293.1 (75.5) | 1185.4 (72.7) |  |
| Yes (F3-F4) | 257 (24.5) | 124 (22) |  |  | 418.6 (24.5) | 446.0 (27.3) |  |
| Diabetes mellitus |  |  | <0.001 |  |  |  | 0.065 |
| No | 1040 (99) | 513 (91.1) |  |  | 1680.8 (98.2) | 1573.2 (96.4) |  |
| Yes | 10 (1) | 50 (8.9) |  |  | 30.9 (1.8) | 58.2 (3.6) |  |
| Hypertension |  |  | <0.001 |  |  |  | 0.840 |
| No | 1023 (97.4) | 517 (91.8) |  |  | 1641.1 (95.9) | 1559.9 (95.6) |  |
| Yes | 27 (2.6) | 46 (8.2) |  |  | 70.6 (4.1) | 71.5 (4.4) |  |
| Duration of antiviral treatment |  |  | <0.001 |  |  |  | 0.845 |
| Never or <6 months | 264 (25.1) | 189 (33.6) |  |  | 440.2 (25.7) | 429.6 (26.3) |  |
| ≥6 months | 786 (74.9) | 374 (66.4) |  |  | 1271.5 (74.3) | 1201.8 (73.7) |  |
| Duration of statins treatment |  |  | 0.010 |  |  |  | 0.941 |
| Never or <1 months | 1031 (98.2) | 540 (95.9) |  |  | 1664.4 (97.2) | 1585.0 (97.2) |  |
| ≥1 months | 19 (1.8) | 23 (4.1) |  |  | 47.3 (2.8) | 46.4 (2.8) |  |
| Duration of metformin treatment |  |  | 0.001 |  |  |  | 0.403 |
| Never or <3 months | 1047 (99.7) | 552 (98) |  |  | 1704.0 (99.5) | 1618.3 (99.2) |  |
| ≥3 months | 3 (0.3) | 11 (2) |  |  | 7.7 (0.5) | 13.1 (0.8) |  |
| Duration of ACEI/ARBs treatment |  |  | <0.001 |  |  |  | 0.988 |
| Never or <6 months | 1038 (98.9) | 538 (95.6) |  |  | 1673.8 (97.8) | 1595.6 (97.8) |  |
| ≥6 months | 12 (1.1) | 25 (4.4) |  |  | 37.9 (2.2) | 35.8 (2.2) |  |
| Duration of low dose aspirin treatment |  |  | 1.000 |  |  |  | 0.652 |
| Never or <3 months | 1047 (99.7) | 562 (99.8) |  |  | 1707.9 (99.8) | 1629.2 (99.9) |  |
| ≥3 months | 3 (0.3) | 1 (0.2) |  |  | 3.8 (0.2) | 2.2 (0.1) |  |
| AFP (ng/ml) |  |  | 0.876 |  |  |  | 0.531 |
| ≤8.1 (Normal) | 840 (80) | 453 (80.5) |  |  | 1345.7 (78.6) | 1244.9 (76.3) |  |
| >8.1 (Elevated) | 210 (20) | 110 (19.5) |  |  | 366.0 (21.4) | 386.5 (23.7) |  |
| Albumin (g/L) | 44.50 (41.12, 46.90) | 45.40 (42.25, 48.00) | <0.001 |  | 44.10 (41.20, 46.80) | 44.20 (40.60, 47.40) | 0.701 |
| Total bilirubin (μmol/L) | 12.95 (9.60, 17.20) | 12.30 (9.45, 16.25) | 0.057 |  | 12.30 (10.00, 16.80) | 12.30 (9.40, 17.10) | 0.620 |
| ALP (U/L) | 71.00 (58.00, 84.00) | 76.00 (63.00, 90.00) | <0.001 |  | 72.00 (61.00, 87.00) | 74.00 (61.00, 89.00) | 0.678 |
| ALT (U/L) | 37.00 (22.25, 77.00) | 40.00 (26.00, 67.00) | 0.361 |  | 32.00 (19.00, 67.00) | 39.00 (24.00, 72.00) | 0.061 |
| AST (U/L) | 32.00 (23.00, 53.00) | 28.00 (22.00, 45.00) | 0.019 |  | 30.00 (21.00, 46.00) | 31.00 (23.00, 52.00) | 0.133 |
| GGT (U/L) | 27.00 (17.00, 49.00) | 34.00 (23.00, 56.50) | <0.001 |  | 28.00 (19.00, 51.00) | 32.00 (21.00, 56.00) | 0.090 |
| Fasting glucose (mmol/L) | 4.79 (4.37, 5.11) | 4.97 (4.59, 5.44) | <0.001 |  | 4.80 (4.51, 5.13) | 4.85 (4.49, 5.29) | 0.198 |
| Triglyceride (mmol/L) | 0.87 (0.69, 1.12) | 1.17 (0.90, 1.57) | <0.001 |  | 0.97 (0.74, 1.37) | 0.98 (0.78, 1.31) | 0.948 |
| Total cholesterol (mmol/L) | 4.47 (3.96, 5.08) | 4.91 (4.23, 5.55) | <0.001 |  | 4.68 (4.07, 5.27) | 4.68 (3.97, 5.31) | 0.444 |
| HDL cholesterol (mmol/L) | 1.30 (1.10, 1.52) | 1.13 (0.96, 1.32) | <0.001 |  | 1.22 (0.99, 1.46) | 1.23 (1.02, 1.47) | 0.576 |
| LDL cholesterol (mmol/L) | 2.75 (2.32, 3.30) | 3.21 (2.70, 3.78) | <0.001 |  | 2.79 (2.34, 3.43) | 2.96 (2.28, 3.50) | 0.503 |
| HBV DNA |  |  | <0.001 |  |  |  | 0.458 |
| <6 log_10_ IU/ml | 562 (53.5) | 362 (64.3) |  |  | 1026.4 (60.0) | 928.2 (56.9) |  |
| ≥6 log_10_ IU/ml | 488 (46.5) | 201 (35.7) |  |  | 685.3 (40.0) | 703.2 (43.1) |  |
| HBeAg |  |  | 0.002 |  |  |  | 0.404 |
| Negative | 533 (50.8) | 331 (58.8) |  |  | 975.0 (57.0) | 871.5 (53.4) |  |
| Positive | 517 (49.2) | 232 (41.2) |  |  | 736.7 (43.0) | 759.9 (46.6) |  |

Data were expressed as number (%) or median (IQR).

NAFLD, non-alcoholic fatty liver disease; IPTW, inverse probability treatment weighting; BMI, body mass index; ACEI, angiotensin-converting enzyme inhibitors; ARBs, angiotensin receptor blockers; AFP, alpha-fetoprotein; ALP, alkaline phosphatase; ALT, alanine aminotransferase; AST, aspartate aminotransferase; GGT, gamma-glutamyl transferase; HDL, high-density lipoprotein; LDL, low-density lipoprotein; HBV, hepatitis B virus; HBeAg, hepatitis B e antigen; IQR, interquartile range.
